# Long‐term patient‐reported outcomes and toxicity following salvage radiotherapy for biochemical recurrence after radical prostatectomy

**DOI:** 10.1002/cncr.70609

**Published:** 2026-09-15

**Authors:** Connor Lynch, Muzamil Arshad, Greeshma Rajeev‐Kumar, Yan Che, Ted A. Skolarus, Parth K. Modi, Stanley L. Liauw

**Affiliations:** ^1^ Department of Radiation Oncology University of Chicago Chicago Illinois USA; ^2^ Department of Statistics University of Chicago Chicago Illinois USA; ^3^ Department of Urology University of Chicago Chicago Illinois USA

**Keywords:** intensity‐modulated radiation therapy, prostate cancer, radiation effects, radiotherapy, quality of life

## Abstract

**Background:**

Salvage radiotherapy (RT) after prostatectomy is common, but studies reporting long‐term follow‐up on patient‐reported outcomes (PROs) and toxicity are limited. The authors report the largest cohort of patients treated with intensity modulated radiotherapy (IMRT) to date assessing patient‐reported quality of life (QOL) and physician‐rated toxicity with long‐term follow‐up.

**Methods:**

PROs were prospectively collected for men undergoing post‐prostatectomy salvage IMRT. Declines in quality of life were defined using established minimum clinically important difference (MCID) thresholds for bowel function (BF), sexual function (SF), urinary incontinence (UI), and urinary obstruction/irritation (UO) domains. Physician‐reported genitourinary (GU) and gastrointestinal (GI) toxicities were recorded with Common Terminology Criteria for Adverse Events v3.0.

**Results:**

Between 2007 and 2019, 317 men were treated to a median dose of 68.4 Gy to the prostate bed with 72% receiving pelvic nodal treatment and 72% receiving androgen deprivation therapy. Median follow‐up was 63 months. The median scores for UI, UO, BF, and SF did not decline more than the MCID at any time point except for the 6‐week bowel score. At 5 years, the percentage of men with QOL decline exceeding MCID for BF, SF, UI, and UO domains were 23%, 25%, 29%, and 31%, respectively. The cumulative incidences of grade 3+ GU and GI toxicity at 5 years were 5.1% and 1.6%, respectively.

**Conclusion:**

Median patient‐reported QOL scores remained largely stable following salvage RT after radical prostatectomy, although clinically meaningful declines occurred in a subset of patients. QOL decline and physician‐rated toxicity were transient in approximately one‐third of affected patients.

## Abstract 2

In this prospective cohort of 317 men treated with salvage intensity‐modulated radiotherapy after radical prostatectomy, median patient‐reported quality‐of‐life scores remained largely stable over long‐term follow‐up. Although a clinically meaningful subset of patients experienced domain‐specific declines, severe physician‐rated toxicity was uncommon.

## INTRODUCTION

Although radical prostatectomy is an effective treatment for localized prostate cancer, patients with high risk features such as pathologic T3 disease or positive margins have a 20%–50% chance of biochemical recurrence following surgery.^1^ Post‐prostatectomy salvage radiotherapy (SRT) for a rising prostate‐specific antigen (PSA) is a standard next‐line treatment for these men. Three trials and a combined meta‐analysis have established that salvage radiotherapy does not compromise efficacy when compared to adjuvant RT, and allows a significant proportion of men to avoid treatment using a PSA threshold of 0.1–0.2 ng/mL for treatment following radical prostatectomy.^2^, ^3^, ^4^, ^5^, ^6^

Despite long‐term follow‐up on these trials, patient‐reported outcomes (PROs) regarding quality of life (QOL) changes are sparsely reported. RADICALS‐RT collected patient‐reported urinary and fecal incontinence scores at 1, 5, and 10 years, but did not report other outcomes.^6^ GETUG‐AFU 17 and TROG 08.03 have not reported PROs, and detailed QOL outcomes from RTOG 0534 are awaited.^7^ Prospectively collected PROs from the LAPPRO trial have been reported comparing 8 years post‐surgery to 1 year post‐surgery, but were restricted to patients who did not receive radiotherapy within 1 year of surgery, which may not reflect the current early‐salvage paradigm.^8^ Finally, one prospective report detailed PROs at over 10 years from radiotherapy but patients were treated with three‐dimensional conformal radiotherapy rather than intensity modulated radiotherapy (IMRT).^9^

We have previously reported detailed patient‐reported outcomes among 199 men with just under 3 years of follow‐up.^10^ Although the previous report included a small number of patients treated with adjuvant rather than salvage radiotherapy, this update is restricted to only patients who received salvage radiotherapy to match current clinical practice. We present our updated analysis with an expanded patient cohort and a median follow‐up of over 5 years to characterize outcomes for patients receiving SRT, assess predictors of patient‐ and physician‐reported toxicity, and provide resources for counseling patients on long‐term outcomes following SRT.

## MATERIALS AND METHODS

### Patient selection and treatment

A prospective cohort of men treated for prostate cancer was followed who received SRT following prostatectomy. All patients had a detectable PSA before beginning radiotherapy and were treated with IMRT. Patients received androgen deprivation therapy (ADT) and elective pelvic nodal radiotherapy at the discretion of the treating physician based on clinical and pathologic characteristics. Prostate bed volumes were contoured according to interdisciplinary consensus guidelines^11^, ^12^ with 6–9 mm anisotropic margins.^13^ Institutional treatment planning goals have been reported previously^14^, ^15^ and included sparing goals for the bladder V70Gy<30%, V65Gy<60%, and V40Gy<80%, as well as rectal V70Gy<20%, V65Gy<40%, and V40Gy<80%. These goals were used to guide initial planning but were not prioritized over target coverage, given the lack of proven association between dose–volume histogram data and toxicity with post‐prostatectomy RT,^15^ and common clinical scenario of significant overlap between the planning target volume and bladder. This study was approved by the University of Chicago institutional review board.

### QOL and toxicity evaluation

Patient‐reported QOL outcomes were collected before treatment and then at each post‐treatment follow‐up until patients were lost to follow‐up or died. Post‐treatment follow‐up occurred at 2 months, then at 6‐ to 12‐month intervals thereafter. Patient‐reported data were recorded prospectively using the Expanded Prostate Cancer Index Composite (EPIC‐26) survey, which assesses urinary incontinence (UI), urinary obstruction/irritation (UO), bowel function (BF), and sexual function.^16^ Responses to individual questions regarding urinary pad usage were also used in this analysis.

For assessing change in QOL relative to pretreatment, the minimum clinically important difference (MCID) was defined for each domain as a decline of 7.5, 6, 5, and 11 for UI, UO, BF, and SF domains, respectively.^17^ Physician‐reported late toxicity was also recorded prospectively using the Radiation Therapy Oncology Group (RTOG) and the National Cancer Institute Common Toxicity Criteria for Adverse Events (CTCAE, v3.0).^18^, ^19^ Late toxicity is defined as occurring at 90 or more days from the end of SRT.

Differences in domain score at the evaluated time points were compared to baseline by a one‐sided paired Wilcoxon ranked sum test with Bonferroni correction for multiple testing. General estimating equation (GEE) models to assess the association between clinical factors and PROs. GEE models were performed using continuous EPIC domain scores as dependent variables. Missing values were excluded. Individual variables were assessed with baseline domain scores and follow‐up time included as independent covariates. These are referred to as “univariable” models for simplicity, although the inclusion of the baseline score does make these multivariable in nature. Baseline domain score and follow‐up time were also included in all multivariable models which included multiple patient and treatment characteristics as independent variables. Fine–Gray subdistribution hazard models were used to assess the association between clinical factors and physician‐rated adverse events. Fine–Gray analyses were performed with death treated as a competing event. Covariates included in multivariable analyses were selected based on clinical relevance and statistically significant (*p* < .05) univariable associations. R version 4.4 was used for all statistical analyses (R core group) with the *tidycmprsk* package used for estimating and plotting cumulative incidence whereas the *geepack* package was used for GEE modeling.^20^, ^21^

## RESULTS

### Patient and treatment characteristics

In total, 317 consecutive patients treated with salvage radiotherapy between 2007 and 2019 met the criteria for inclusion. Baseline clinical characteristics and treatment parameters are summarized in Table 1. Median follow‐up from the end of radiotherapy treatment was 63 months. The median age was 65. Most patients had pT3a or greater disease (71%) and most had grade group 3 disease or greater (62.2%). Radiation was delivered at a median of 20 months from the time of prostatectomy to the prostate bed (median dose, 68 Gy) with or without pelvic lymphatics (72%, median, 50.4 Gy). ADT was delivered in 72% of men for a median duration of 4 months.

**TABLE 1:** Patient clinical and pathologic characteristics.

Characteristic	*N* = 317^a^
Age, years	65 (60–70)
Race, No. (%)	
Asian	4 (1.3)
Black	89 (29)
Hispanic	16 (5.2)
White	200 (65)
Unknown	8
BMI	28.8 (26.1–32.1)
Tobacco use, No. (%)	
Never	148 (47)
Previous	129 (41)
Current	39 (12)
Unknown	1
Gleason grade group, No. (%)	
1	23 (7.3)
2	96 (30)
3	85 (27)
4	26 (8.2)
5	87 (27)
Pathologic T stage, No. (%)	
T2	93 (29)
T3a	128 (41)
T3b	93 (29)
T4	2 (0.6)
Unknown	1
Node‐positive	81 (26%)
Pre‐RT PSA	0.30 (0.17–0.73)
Time from RP to RT (months)	20 (9–48)
Pelvic nodal RT delivered	229 (72%)
Radiotherapy dose (Gy)	68.40 (68.00–68.40)
ADT delivered	229 (72%)
ADT duration (months)	4 (4–12)
Rectum V70Gy(%)	5.6 (2.0–9.6)
Rectum V65Gy(%)	21 (16–27)
Rectum V60Gy(%)	27 (21–34)
Rectum V50Gy(%)	37 (30–46)
Rectum V40Gy(%)	49 (39–63)
Rectum V30Gy(%)	72 (58–87)
Rectum mean dose (Gy)	43 (39–48)
Bladder V70Gy(%)	16 (6–24)
Bladder V65Gy(%)	46 (37–56)
Bladder V60Gy(%)	52 (42–61)
Bladder V50Gy(%)	62 (51–70)
Bladder V40Gy(%)	73 (60–80)
Bladder V30Gy(%)	87 (73–98)
Bladder mean dose (Gy)	55 (48–58)

Abbreviations: ADT, androgen deprivation therapy; BMI, body mass index; PSA, prostate‐specific antigen; RT, radiotherapy; RP, radical prostatectomy.

^a^ Median (Q1, Q3); No. (%).

### Patient‐reported QOL

**FIGURE 1:**
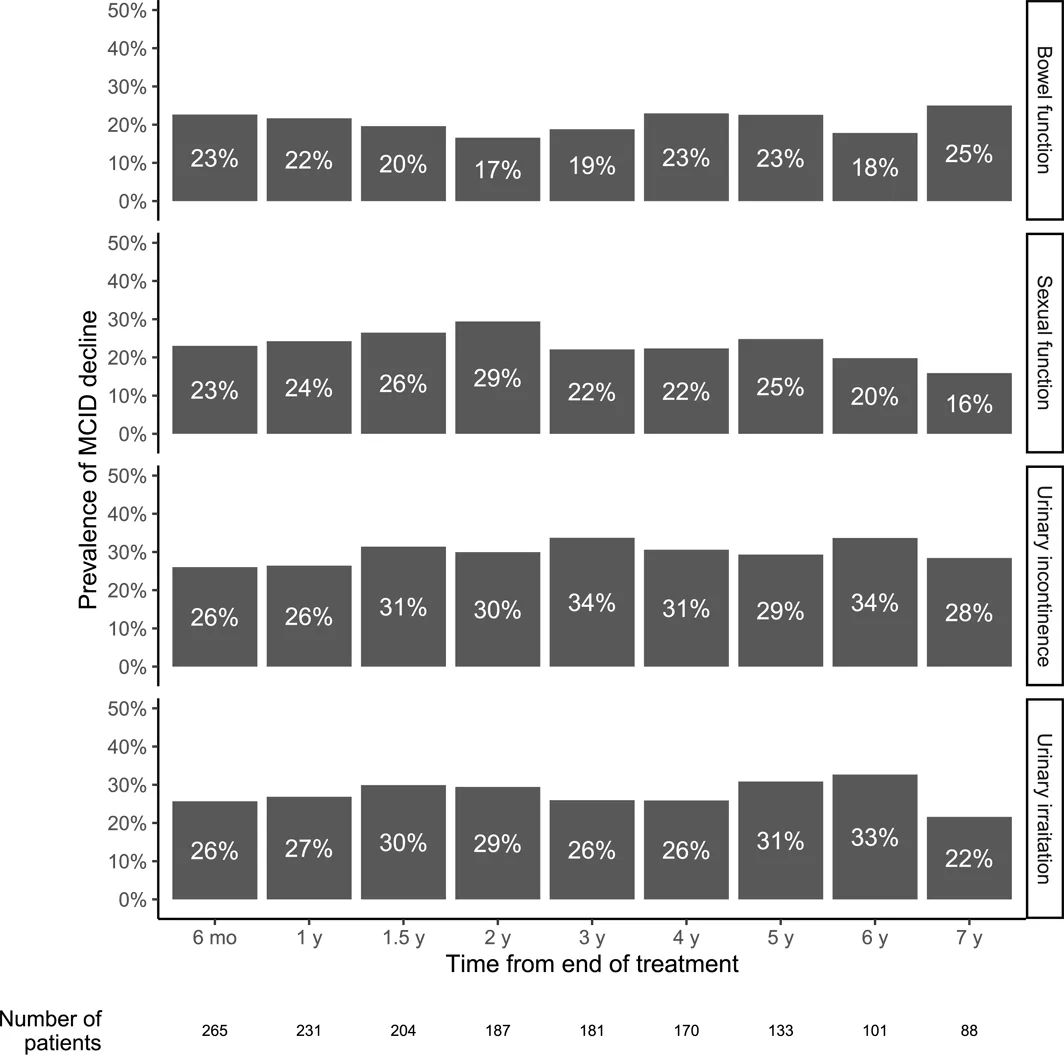
Prevalence of quality‐of‐life decline exceeding the MCID at each follow‐up time point. Bars represent the proportion of evaluable patients at each individual time point whose EPIC domain score declined beyond the established MCID threshold relative to baseline. Values represent point prevalence and should not be interpreted as cumulative incidence. MCID indicates minimum clinically important difference; QOL, quality of life.

**FIGURE 2:**
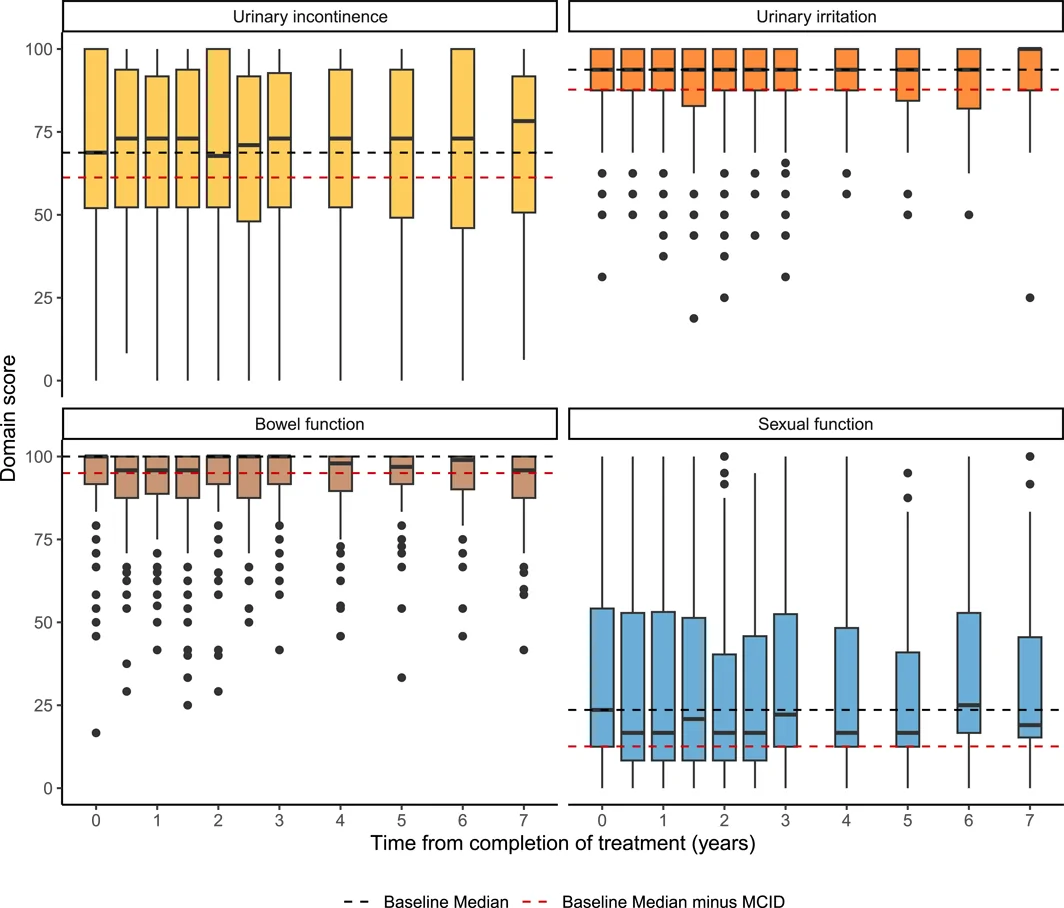
EPIC domain scores over time. Boxplots demonstrate the distribution of EPIC domain scores at each follow‐up time point. Year 0 represents the baseline post‐prostatectomy, pre‐radiotherapy assessment. Higher scores indicate better function. The black dashed line represents the baseline median domain score for the cohort. The red dashed line represents a decline greater the MCID from the baseline median score. EPIC indicates Expanded Prostate Cancer Index Composite; MCID, minimum clinically important difference.

The rates of decline in patient‐reported QOL exceeding the MCID through 7 years are reported in Figure 1, and patient reported symptom distress is reported in Table S1. At 5 years, rates of QOL decline exceeding MCID for BF, SF, UI, and UO domains were 23%, 25%, 29%, and 31%, respectively. The rate of multi‐domain decline (decline exceeding MCID in all four domains) was 2% at 5 years, with half of these patients experiencing declines at least twice the magnitude of the established MCID threshold in all domains. The incidences of QOL decline exceeding MCID at any time through 5 years of follow‐up for the BF, SF, UI, and UO domains were 45%, 39%, 49%, and 55%, respectively. Rates of MCID decline exceeding twice the established threshold are shown in Figure S1. By the time of last follow‐up, 33%, 24%, 19%, and 29% of men who experienced a decline in QOL in these domains had recovered such that their QOL scores no longer declined beyond the MCID. EPIC domain scores over time are displayed in Figure 2. In cases where the EPIC scores were significantly different from baseline after multiple testing correction, the difference between the median domain score at each time point and the median baseline domain score did not exceed the MCID, suggesting that median patient‐reported function remained relatively stable over time despite variability at the individual patient level.

### Urinary pad use

Of 274 men who answered the survey question on pad usage at baseline and on at least one subsequent visit, 62% experienced no change in pad usage at their last follow‐up. Increased pad usage was seen in 21% of patients whereas the remainder (17%) had decreased pad usage. Pad usage over time is further characterized in Figure 3A, which displays pad usage over time organized by baseline pad usage. At 5 years, approximately 78% of men requiring no pads at baseline remained without need for pads. Approximately 30% of men requiring two or more pads at baseline experienced improvement.

FIGURE 3Patient‐reported outcomes stratified by baseline function. Labels for percentage smaller than 6% are omitted for display purposes. Bolded numbers below each bar show the total number of patients at each time point. (A) Demonstrates urinary pad usage at follow‐up stratified by baseline pad usage. (B) Shows erectile function at follow‐up, stratified by baseline erectile function. Function was counted as the maximum rating provided for erectile function either with or without medication or assistive devices.

**Figure:**
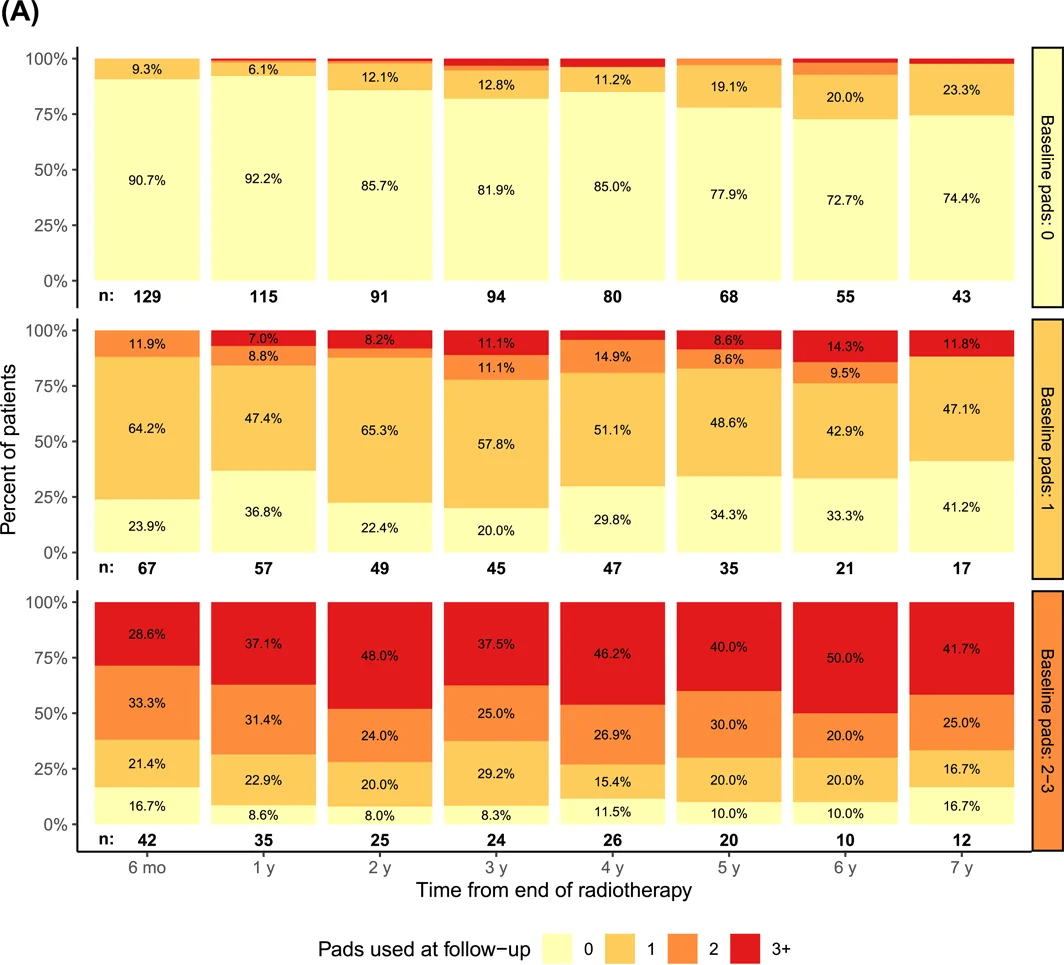

**Figure:**
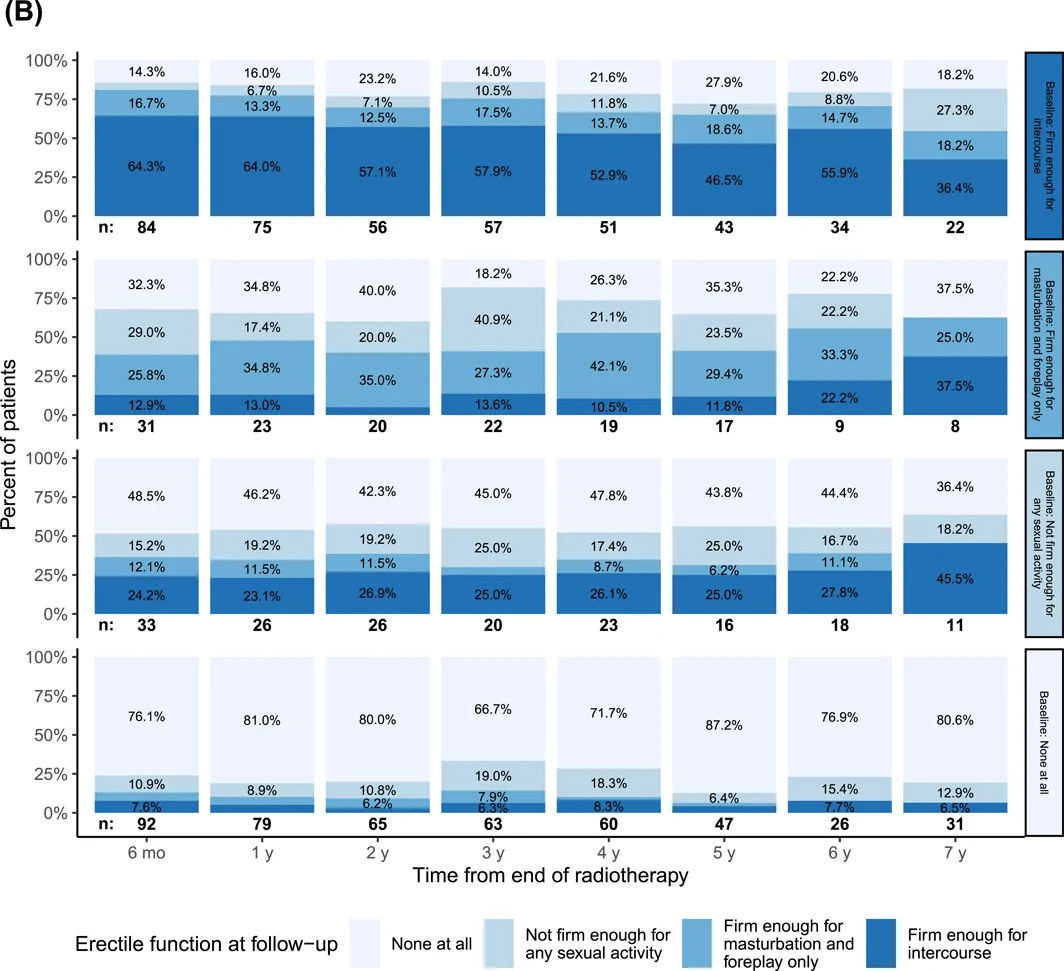

### Sexual aids and erectile function

Before radiotherapy, 29% of men reported experiencing erections that were adequate for penetrative intercourse without medications or devices. With medications and/or devices, the proportion was 31%. Most men had tried at least one intervention for erectile dysfunction before beginning radiotherapy. Phosphodiesterase E5 (PDE5) inhibitors such as sildenafil were the most commonly tried intervention (72%) with 55% of those men reporting improvement in erectile function. The next most common intervention attempted was intracavernosal injection (13%) with 81% of those men reporting improvement in erectile function. Other pre‐radiotherapy interventions included vacuum pump devices (10%), implanted devices (5%), and intraurethral suppositories (4%). At 5 years, 27% of patients reported improvement in erectile function attributable to medication or devices. Of those who reported trying PDE5 inhibitors, 39% experienced a benefit. Intracavernosal injection remained the second‐most tried therapy at 5 years with 56% of men reporting a benefit. Erectile function over time is shown in Figure 3B. Approximately 47% of men with baseline erections firm enough for intercourse maintained this level of sexual health at 5 years.

### Clinical and dosimetric predictors of patient‐reported toxicity

Predictors of patient‐reported QOL were assessed with generalized estimating equations modeling. Significant univariable predictors (*p* < .05) (Tables S2–S4) were included in a multivariable model for each domain. The multivariable results are provided in Table 2. Results of univariable testing are provided in Tables S2–S5. This analysis identified long‐term ADT use and the percent of the bladder receiving 70 Gy (bladder V70Gy) as predictors of urinary incontinence. Greater urinary irritation was associated with smoking at the time of treatment. Time from surgery to RT was associated with worse bowel function, whereas inclusion of pelvic lymph nodes was not. Decline in sexual function was associated with greater age at treatment and diabetes.

**TABLE 2:** General estimating equation model results for predictors of patient‐reported outcomes.

Group	Characteristic	*β*	95% CI	*p*
Urinary incontinence	Baseline urinary incontinence score	0.68	0.60−0.75	<.001
	Time of follow‐up	−0.80	−1.3 to −0.32	.001
	Age at treatment	−0.25	−0.54 to 0.03	.079
	Time from RP to RT (months)	−0.05	−0.10 to 0.00	.076
	Bladder V70 Gy (%)	−0.29	−0.47 to −0.10	.003
Urinary irritation	Baseline urinary irritation score	0.41	0.25−0.57	<.001
	Time of follow‐up	−0.28	−0.49 to −0.06	.011
	Age at treatment	−0.06	−0.22 to 0.10	.5
	BMI at treatment	−0.04	−0.20 to 0.13	.7
	Race			
	White	—	—	
	Black	−2.9	−5.6 to −0.28	.030
	Other	2.3	−1.5 to 6.0	.2
	Time from RP to RT (months)	−0.02	−0.04 to 0.01	.2
	Smoking history			
	Never	—	—	
	Previous	−0.37	−2.5 to 1.8	.7
	Current	−4.8	−9.1 to −0.38	.033
Bowel function	Baseline bowel function score	0.63	0.52−0.74	<.001
	Time of follow‐up	0.04	−0.16 to 0.24	.7
	Time from RP to RT (months)	−0.03	−0.05 to 0.00	.025
Sexual function	Baseline sexual function score	0.63	0.55−0.71	<.001
	Time of follow‐up	0.09	−0.37 to 0.55	.7
	Age at treatment	−0.28	−0.53 to −0.03	.028
	Androgen deprivation therapy			
	No	—	—	
	Yes	−4.2	−8.6 to 0.18	.060
	Diabetes mellitus			
	Nondiabetic	—	—	
	Diabetic	−6.7	−12 to −0.97	.022

*Notes:* General estimating equation multivariable model output. Higher EPIC domain scores reflect better patient‐reported outcomes. Positive beta coefficients reflect a positive association with score such that as the value for that characteristic increases, the domain score reported is expected to increase.

Abbreviations: CI, confidence interval; EPIC, Expanded Prostate Cancer Index Composite; RP, radical prostatectomy; RT, radiotherapy; VXX%Gy, volume of structure receiving XX (e.g., 70) Gy.

### Physician‐rated toxicity

**FIGURE 4:**
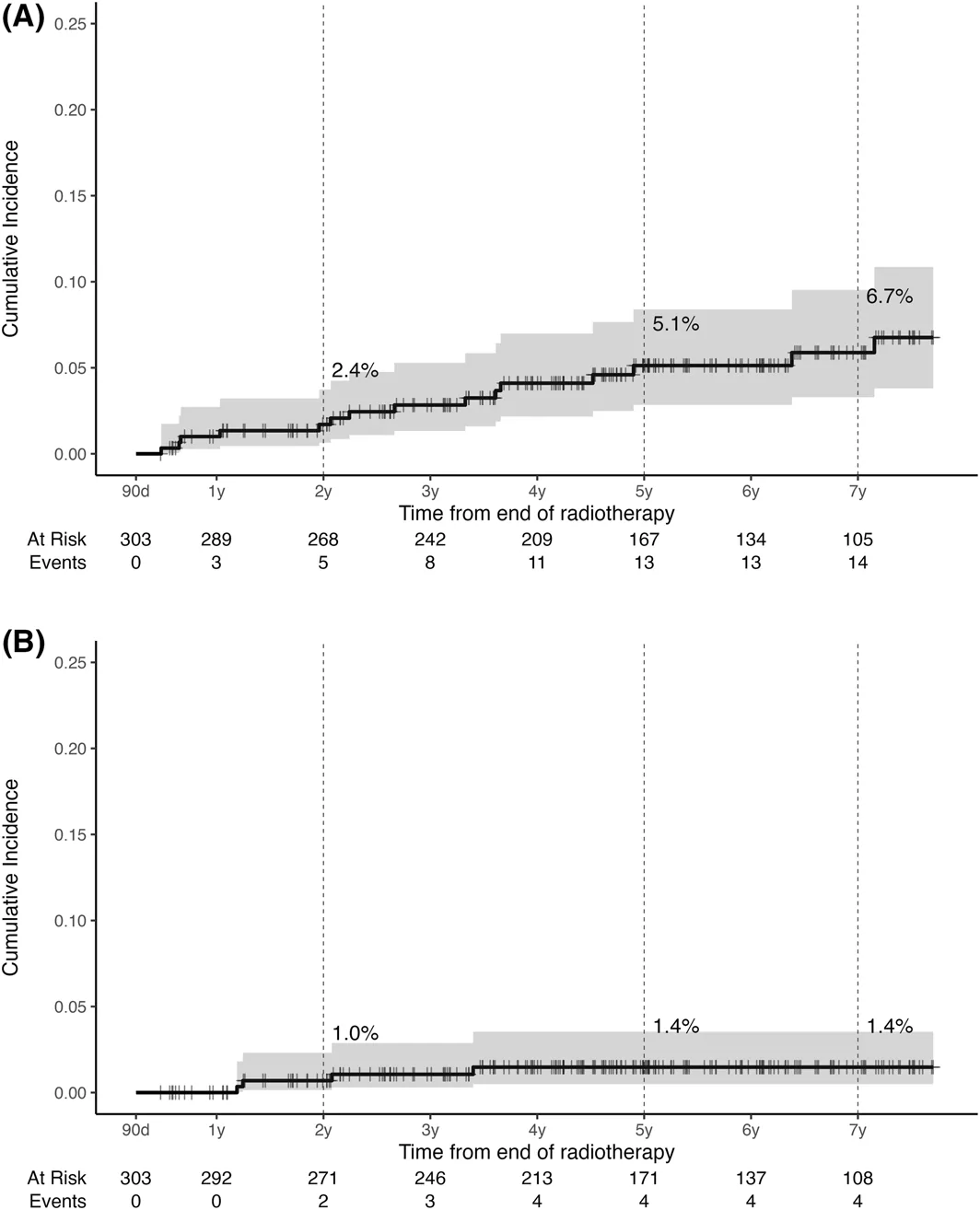
Cumulative incidence of physician rated grade 3 or GU and GI adverse events. Shaded regions represent 95% confidence intervals. Numbers at risk are displayed below each panel. GI indicates gastrointestinal; GU indicates genitourinary.

**FIGURE 5:**
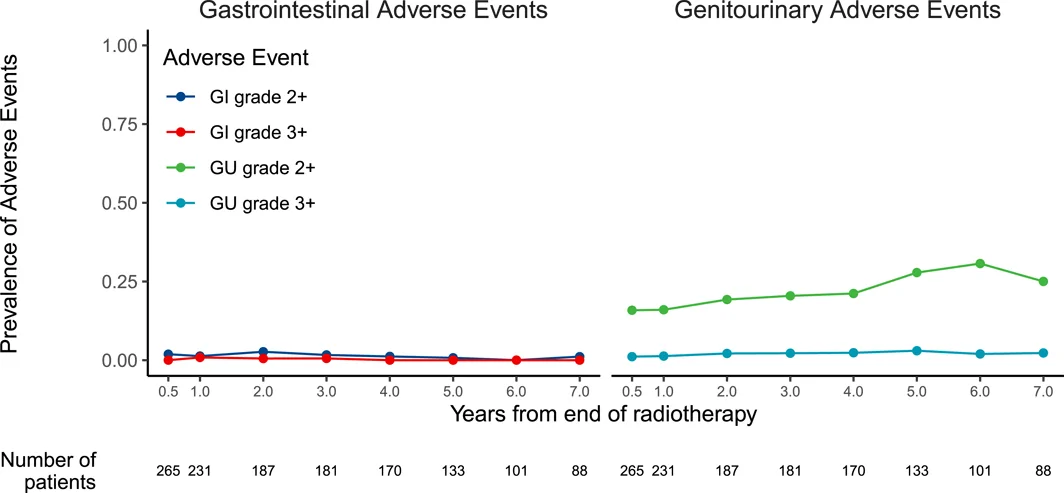
The prevalence of physician‐rated grade 2+ and grade 3+ GI and GU adverse events at each follow‐up time point. Values represent point prevalence among evaluable patients rather than cumulative incidence. GI indicates gastrointestinal; GU indicates genitourinary.

The cumulative incidences of physician‐rated CTCAE grade 3+ GU and GI AEs are plotted in Figure 4A,B, respectively, with estimates provided for incidences at 2, 5, and 7 years. The estimated cumulative incidence of grade 3 GU AEs was 1.7%, 5.1%, and 6.7% at 2, 5, and 7 years, respectively. Of the 15 grade 3 events recorded: five were incontinence, five were cystitis, and five were obstruction. Fourteen events are identified in Figure 4A because grade 3 cystitis and obstruction were diagnosed in the same patient on the same visit and were counted as one. The most common grade 2+ GU adverse event was urinary incontinence with an overall incidence of 25% over the total study follow‐up period. Grade 2+ cystitis, obstruction, and frequency occurred in 9%, 9%, and 7% of patients over the follow‐up period, respectively. Grade 3+ GI toxicity was estimated to be less than 2% through 7 years of follow‐up. There were three cases of grade 3 rectal bleeding and one case of grade 3 proctitis. Diarrhea was the most common grade 2+ GI adverse event at 4% over the total study follow‐up period. There were no grade 4 or 5 adverse GI or GU events recorded. The prevalence of grade 2+ and grade 3+ GU and GI AEs through 7 years of follow‐up is plotted in Figures 4 and 5.

Because grade 3+ events were relatively uncommon, multivariable Fine–Gray analyses were performed using grade 2+ toxicity end points to provide more stable estimates of predictors of toxicity. Multivariable model results for predictors of physician‐rated toxicity are provided in Table 3. Univariable results are given in Tables S6 and S7. Increasing bladder V70Gy(%), anticoagulation use, and Black race were associated with higher rates of physician related toxicity. No patient or treatment characteristic was significantly associated with risk for physician‐rated GI toxicity on multivariable analysis. Although patient race was significantly associated with toxicity on univariable analysis, the category associated with reduced GI toxicity (“Other”) had a small number of patients and no events. Given challenge in interpreting such a finding in a broad category with few patients, it was excluded from the multivariable analysis. Treatment of the pelvis was not associated with greater toxicity than prostate bed alone.

**TABLE 3:** Predictors of grade 2+ CTCAE genitourinary and gastrointestinal toxicity by multivariable Fine–Gray analysis.

Group	Characteristic	*N*	HR	95% CI	*p*
Genitourinary toxicity	Race				
	White	190	—	—	
	Black	84	1.55	1.01–2.37	.044
	Other	18	1.26	0.50–3.12	.6
	Bladder V70Gy(%)	292	1.03	1.01–1.05	.005
	Anticoagulation use				
	No	273	—	—	
	Yes	19	2.24	1.13–4.41	.020
	Aspirin use				
	No	176	—	—	
	Yes	116	1.55	1.02–2.34	.040
Gastrointestinal toxicity	Time from RP to RT (months)	302	1.01	1.00–1.02	.054
	Treatment volume				
	Prostate bed	83	—	—	
	Pelvis	219	0.62	0.21–1.85	.4

Abbreviations: CI, confidence interval; CTCAE, Common Terminology Criteria for Adverse Events; HR, hazard ratio, RT, radiotherapy; VXX%Gy, volume of structure receiving XX (e.g., 70) Gy.

## DISCUSSION

We report a comprehensive analysis of prospectively recorded long‐term patient‐ and physician‐reported outcomes in 317 men treated at a single center. This analysis combines commonly used metrics such as the MCID for assessing changes in patient‐reported symptoms, as well as plain‐English summaries of outcomes that matter to patients including urinary and erectile function. Although the overall incidence of decline in QOL exceeding MCID ranges from 39% to 55% depending on the domain, up to a third of these patients experienced improvement at last follow‐up. The lower prevalence of QOL decline exceeding MCID (16%–34% depending on the domain) demonstrates that effects on QOL are transient for many patients. Cumulative incidence of grade 3+ GU and GI toxicity were low out to 5 years of follow‐up at 5.1% and 1.4%, respectively. These findings offer reassurance that SRT with or without ADT and pelvic RT results in low rates of significant QOL decline or toxicity, even years after treatment.

An important consideration when interpreting these findings is that baseline assessments were obtained after radical prostatectomy but before salvage radiotherapy. Accordingly, baseline scores represent post‐surgical function rather than pretreatment function. Longitudinal changes therefore likely reflect a combination of radiotherapy effects, ongoing post‐prostatectomy recovery, androgen deprivation therapy, aging, and comorbidities. Although the present study was designed to characterize outcomes following salvage radiotherapy, the independent contribution of radiotherapy to long‐term functional changes cannot be fully isolated.

Our PRO results are generally consistent with data from prospective trials and other studies using the EPIC‐26 instrument. The phase 3 NRG‐GU003 trial examining hypofractionated versus conventionally fractionated SRT identified changes in EPIC‐26 urinary and bowel domain scores at 2 years among conventionally fractionated SRT patients were –4.07 and –1.41, respectively, comparable to the –1.68 and –1.69 observed in our cohort. Longer term outcomes are awaited.^22^ The RAVES trial secondary analysis reported persistent urinary incontinence domain decline exceeding the MCID in 24.5% of patients, a figure consistent with the 28% we observed at 5 years.^4^ Although the RADICALS‐RT trial demonstrated a decline in patient‐reported quality of life at 1‐year, this difference was not sustained at 5 years, consistent with our findings.^2^ Another 10‐year report identified continued late QOL declines at up to 13 years post‐treatment. We did not observe a similar trend, though this may reflect differences in technique as their cohort was treated with 3D‐conformal RT rather than IMRT, which has been associated with reduced late GI toxicity.^9^, ^23^

Several unique aspects of our data merit further discussion. Although approximately 20% of men experienced QOL declines beyond the MCID at any given time point, median EPIC domain scores remained stable throughout follow‐up, suggesting that the typical patient does not experience progressive late decline despite meaningful variability among individual patients. The detailed erectile function and sexual aid data in this cohort are relatively uncommon in the postoperative RT literature. Stratifying by baseline function, we show that up to a third of men whose erectile function was inadequate for sexual activity may still see improvement following SRT. These data underscore the importance of comprehensive sexual rehabilitation counseling in this population. Additionally, the absence of significantly worse PROs among the 72% of men who received pelvic nodal RT is noteworthy given the larger irradiated volume. Our physician‐reported toxicity analysis similarly found no increased risk for GU or GI toxicity with pelvic nodal treatment, consistent with the 5‐year data from RTOG 0534, which also found no difference in physician‐reported GI toxicity in patients treated with pelvic nodal RT. Finally, with a median follow‐up of 5 years, these data provide reassuring intermediate‐term follow‐up regarding severe toxicity. However, continued surveillance remains important because late toxicity may continue to accumulate beyond 10 years after treatment.^24^ GU toxicity curves do increase over time as described in other reports, but the rate of severe toxicity was 5% at 5 years. Grade 2+ GU toxicity, which is comprised of frequency, incontinence, and cystitis/hematuria,^25^ does occur at a substantially higher rate of >25% at 5 years. Whether serious late effects were more limited due to the conventional fractionation is uncertain, but may become clearer as hypofractionated RT studies mature. The increasing risk of grade 2+ GU toxicity over time justifies the development of trials evaluating dose de‐escalation, given that bladder dose is correlated with GU toxicity, and contemporary patients lacking identifiable disease on positron emission tomography and magnetic resonance imaging referred for early salvage RT may not require doses of 66–68 Gy.

Regarding predictors of QOL and physician‐reported outcomes, multivariable analysis identified increasing bladder V70Gy as associated with worse urinary incontinence and GU toxicity, suggesting that patients receiving a boost dose for gross disease may face greater risk. As the SAKK 09/10 trial demonstrated, there is no benefit to routinely dose‐escalating to 70 Gy for post‐prostatectomy patients without gross prostate bed disease.^26^ This also highlights the need to be mindful of the location of dosimetric hotspots in standard‐dose plans. Current smoking was associated with greater urinary irritation, consistent with tobacco’s known activity as a bladder irritant,^27^ whereas age and diabetes predicted sexual function decline, both established risk factors for erectile dysfunction independent of RT.^28^ GU toxicity was associated with Black race, bladder V70 Gy(%), and anticoagulation use, as we have previously reported.^10^ Although our previous analysis of fewer patients identified body mass index (BMI) as associated with multiple outcomes,^10^ the current analysis did not. Although multiple effective medications for weight loss have been introduced since the time of the last publication and there has been an increasing emphasis on lifestyle interventions in cancer patients, we hypothesize that the difference in this analysis is likely due to increased heterogeneity of the cohort. The impact of weight loss and other lifestyle interventions on patient QOL merits further study.

This study has several limitations. These data were collected at a single institution. Additionally, missing follow‐up data at later time points reduced statistical power and may have introduced selection bias. Although GEE analyses permit inclusion of patients with incomplete longitudinal observations, follow‐up attrition increased over time, particularly beyond 5 years. Patients with persistent symptoms may have been either more or less likely to remain engaged in long‐term follow‐up, potentially influencing estimates of late outcomes. Accordingly, results from later time points should be interpreted with appropriate caution. Despite these limitations, our study has notable strengths including its prospective design, the use of validated PRO instruments alongside physician‐rated AEs, a large cohort size, and unique data on symptom distress and sexual aid utilization that are infrequently reported in the SRT literature. Future studies incorporating longer follow‐up, multi‐institutional cohorts, and hypofractionation regimens, will be valuable in further defining long‐term QOL after salvage treatment.

In conclusion, this prospective cohort of men treated with salvage IMRT following radical prostatectomy demonstrated generally stable median patient‐reported quality‐of‐life scores during long‐term follow‐up, although clinically meaningful declines occurred in a subset of patients. Severe physician‐rated toxicity was uncommon. These findings provide contemporary long‐term outcome data that may assist clinicians in counseling patients regarding expectations after salvage radiotherapy.

## AUTHOR CONTRIBUTIONS

**Connor Lynch**: Data curation; formal analysis; investigation; methodology; writing—original draft; writing—review and editing. **Muzamil Arshad**: Writing—original draft; writing—review and editing. **Greeshma Rajeev‐Kumar**: Investigation; writing—review and editing. **Yan Che**: Data curation; formal analysis; methodology. **Ted A. Skolarus**: Writing—review and editing. **Parth K. Modi**: Writing—review and editing. **Stanley L. Liauw**: Conceptualization; investigation; methodology; project administration; supervision; writing—review and editing.

## CONFLICT OF INTEREST STATEMENT

The authors declare no conflicts of interest.

## Supporting information

Supporting Information S1

## Data Availability

The data that support the findings of this study are available on request from the corresponding author. The data are not publicly available due to privacy or ethical restrictions.
