# Supplementary material for: Long‐term patient‐reported outcomes and toxicity following salvage radiotherapy for biochemical recurrence after radical prostatectomy
Source: Cancer. 2026 Sep 15;132(18):e70609. doi: 10.1002/cncr.70609 (PMC13579068; doi:10.1002/cncr.70609)
Supplement: Supplementary file 1 [file CNCR-132-e70609-s001.docx]

Supplemental Tables and Figures

Supplemental Table 1. Patient-reported outcomes, proportion of men reporting moderate or severe distress for the individual questions that comprise the global domain scores.

| **Characteristic** | **baseline**  N = 317^1^ | **6 mo**  N = 265^1^ | **1 y**  N = 231^1^ | **1.5 y**  N = 204^1^ | **2 y**  N = 187^1^ | **2.5 y**  N = 145^1^ | **3 y**  N = 181^1^ | **4 y**  N = 170^1^ | **5 y**  N = 133^1^ | **6 y**  N = 101^1^ | **7 y**  N = 88^1^ |
| --- | --- | --- | --- | --- | --- | --- | --- | --- | --- | --- | --- |
| Urinary irritation or obstruction |  |  |  |  |  |  |  |  |  |  |  |
| Dysuria | 3 (1.0%) | 4 (1.6%) | 0 (0%) | 2 (1.0%) | 2 (1.1%) | 0 (0%) | 2 (1.2%) | 0 (0%) | 0 (0%) | 0 (0%) | 1 (1.3%) |
| Hematuria | 0 (0%) | 2 (0.8%) | 4 (1.8%) | 6 (3.1%) | 3 (1.7%) | 1 (0.7%) | 2 (1.2%) | 1 (0.6%) | 3 (2.3%) | 3 (3.2%) | 2 (2.6%) |
| Weak stream | 15 (5.1%) | 7 (2.8%) | 8 (3.6%) | 9 (4.7%) | 10 (5.6%) | 4 (2.9%) | 8 (4.7%) | 6 (3.7%) | 2 (1.6%) | 4 (4.2%) | 1 (1.3%) |
| Nocturia | 36 (12%) | 42 (17%) | 35 (16%) | 31 (16%) | 34 (19%) | 14 (10%) | 32 (19%) | 22 (14%) | 16 (13%) | 7 (7.7%) | 7 (9.0%) |
| Urinary frequency | 34 (12%) | 34 (13%) | 24 (11%) | 27 (14%) | 27 (15%) | 15 (11%) | 23 (13%) | 20 (12%) | 16 (12%) | 10 (11%) | 9 (12%) |
| Urinary incontinence |  |  |  |  |  |  |  |  |  |  |  |
| Urinary leakage | 43 (15%) | 37 (15%) | 35 (16%) | 28 (14%) | 21 (12%) | 23 (17%) | 33 (19%) | 30 (18%) | 22 (17%) | 16 (17%) | 12 (16%) |
| Urine leaking ≥1 time per day | 143 (48%) | 106 (41%) | 94 (42%) | 86 (44%) | 88 (49%) | 62 (45%) | 74 (42%) | 75 (46%) | 66 (51%) | 37 (39%) | 33 (42%) |
| Frequent dribbling | 51 (17%) | 40 (16%) | 40 (18%) | 30 (15%) | 33 (18%) | 30 (22%) | 38 (22%) | 35 (21%) | 20 (16%) | 21 (22%) | 18 (23%) |
| Any urinary pad use | 135 (46%) | 104 (41%) | 83 (37%) | 76 (39%) | 80 (45%) | 57 (41%) | 78 (44%) | 74 (45%) | 57 (44%) | 41 (43%) | 32 (41%) |
| Urinary pads per day ≥2 | 53 (18%) | 35 (14%) | 36 (16%) | 30 (15%) | 28 (16%) | 25 (18%) | 32 (18%) | 34 (21%) | 23 (18%) | 18 (19%) | 12 (15%) |
| Overall urinary problem (moderate or severe) | 35 (12%) | 28 (11%) | 29 (13%) | 25 (13%) | 27 (15%) | 20 (14%) | 25 (14%) | 26 (16%) | 13 (10%) | 12 (13%) | 10 (13%) |
| Bowel function |  |  |  |  |  |  |  |  |  |  |  |
| Bowel urgency | 10 (3.4%) | 10 (3.9%) | 9 (4.1%) | 14 (7.1%) | 10 (5.5%) | 5 (3.6%) | 5 (2.8%) | 6 (3.6%) | 5 (3.9%) | 3 (3.2%) | 4 (5.3%) |
| Bowel urgency ≥1 time per day | 25 (8.5%) | 18 (7.0%) | 18 (8.1%) | 15 (7.7%) | 18 (9.9%) | 12 (8.7%) | 14 (7.8%) | 14 (8.5%) | 9 (7.0%) | 8 (8.4%) | 4 (5.2%) |
| Loose stools ≥ half the time | 31 (11%) | 31 (12%) | 31 (14%) | 28 (14%) | 20 (11%) | 11 (8.0%) | 16 (8.9%) | 23 (14%) | 11 (8.6%) | 11 (12%) | 11 (14%) |
| Bowel frequency | 4 (1.4%) | 8 (3.1%) | 7 (3.2%) | 10 (5.2%) | 4 (2.2%) | 4 (2.9%) | 1 (0.6%) | 4 (2.4%) | 4 (3.1%) | 0 (0%) | 2 (2.7%) |
| Fecal incontinence | 3 (1.0%) | 3 (1.2%) | 3 (1.4%) | 3 (1.6%) | 2 (1.1%) | 2 (1.4%) | 1 (0.6%) | 3 (1.8%) | 2 (1.6%) | 2 (2.2%) | 3 (3.9%) |
| Bloody stools | 4 (1.4%) | 7 (2.7%) | 5 (2.3%) | 3 (1.5%) | 1 (0.6%) | 4 (2.9%) | 3 (1.7%) | 3 (1.8%) | 1 (0.8%) | 0 (0%) | 2 (2.6%) |
| Bloody stools ≥ half the time (last 4 weeks) | 5 (1.7%) | 2 (0.8%) | 3 (1.4%) | 4 (2.0%) | 4 (2.2%) | 0 (0%) | 4 (2.2%) | 0 (0%) | 2 (1.6%) | 0 (0%) | 0 (0%) |
| Rectal pain | 1 (0.3%) | 1 (0.4%) | 1 (0.5%) | 2 (1.0%) | 1 (0.6%) | 1 (0.7%) | 1 (0.6%) | 1 (0.6%) | 1 (0.8%) | 0 (0%) | 0 (0%) |
| Overall bowel problem | 8 (2.7%) | 11 (4.3%) | 12 (5.4%) | 10 (5.1%) | 5 (2.8%) | 4 (2.9%) | 8 (4.5%) | 6 (3.7%) | 5 (3.9%) | 2 (2.1%) | 3 (3.9%) |
| Sexual function |  |  |  |  |  |  |  |  |  |  |  |
| Erections (none or poor quality) | 199 (68%) | 176 (70%) | 146 (70%) | 132 (71%) | 125 (72%) | 99 (74%) | 121 (72%) | 115 (75%) | 94 (78%) | 59 (66%) | 49 (77%) |
| Difficulty with orgasm (none or poor quality) | 152 (52%) | 150 (61%) | 124 (59%) | 113 (63%) | 112 (67%) | 81 (62%) | 99 (60%) | 94 (62%) | 79 (66%) | 46 (52%) | 38 (58%) |
| Erections not firm | 206 (69%) | 180 (71%) | 162 (75%) | 144 (75%) | 139 (78%) | 105 (79%) | 125 (72%) | 125 (77%) | 99 (77%) | 65 (70%) | 63 (82%) |
| Erections not reliable (< half of times desired) | 196 (66%) | 170 (69%) | 152 (71%) | 134 (71%) | 133 (76%) | 99 (74%) | 121 (70%) | 117 (72%) | 92 (75%) | 63 (68%) | 54 (73%) |
| Sexual function last 4 weeks (poor or very poor) | 192 (66%) | 177 (71%) | 143 (66%) | 134 (69%) | 128 (75%) | 98 (73%) | 117 (68%) | 119 (73%) | 92 (74%) | 51 (59%) | 56 (73%) |
| Overall sexuality problem | 125 (43%) | 111 (44%) | 83 (38%) | 70 (37%) | 71 (42%) | 54 (40%) | 61 (36%) | 57 (35%) | 47 (38%) | 22 (24%) | 23 (30%) |
| ^1^n (%) | | | | | | | | | | | |

Supplemental Figure 1. Percentage of patients with decline exceeding twice the magnitude of the established MCID threshold by timepoint

Supplemental table 2. Univariable general estimating equation analysis for bowel function

| **Characteristic** | **N** | **Beta** | **95% CI** | **p-value** | test |
| --- | --- | --- | --- | --- | --- |
| Hemoglobin | 1,602 | 0.25 | -0.65, 1.1 | 0.6 | bowel_sum~hg_count |
| White blood cell count | 1,599 | 0.32 | -0.13, 0.76 | 0.2 | bowel_sum~wbc_count |
| Platelet count | 1,602 | 0.00 | -0.02, 0.02 | 0.9 | bowel_sum~plt_count |
| Rectum V70Gy(%) | 1,768 | -0.14 | -0.36, 0.09 | 0.2 | bowel_sum~rectum_v70_p |
| Rectum V65Gy(%) | 1,768 | -0.02 | -0.12, 0.09 | 0.8 | bowel_sum~rectum_v65_p |
| Rectum V60Gy(%) | 1,441 | -0.03 | -0.12, 0.06 | 0.5 | bowel_sum~rectum_V60_p |
| Rectum V50Gy(%) | 1,441 | -0.02 | -0.09, 0.05 | 0.6 | bowel_sum~rectum_v50_p |
| Rectum V40Gy(%) | 1,768 | 0.00 | -0.05, 0.05 | >0.9 | bowel_sum~rectum_v40_p |
| Rectum mean dose (Gy) | 1,441 | -0.05 | -0.17, 0.08 | 0.5 | bowel_sum~rectum_mean |
| Age | 1,783 | 0.02 | -0.13, 0.16 | 0.8 | bowel_sum~age |
| Time from RP to RT (mo) | 1,783 | -0.03 | -0.05, 0.00 | 0.026 | bowel_sum~rp_rt_time |
| Anticoagulation use | 1,783 |  |  |  | bowel_sum~anticoag |
| No |  |  |  |  | bowel_sum~anticoag |
| Yes |  | -1.8 | -6.4, 2.8 | 0.4 | bowel_sum~anticoag |
| Aspirin Use | 1,783 |  |  |  | bowel_sum~aspirin |
| No |  |  |  |  | bowel_sum~aspirin |
| Yes |  | -1.2 | -3.1, 0.65 | 0.2 | bowel_sum~aspirin |
| Diabetes mellitus | 1,768 |  |  |  | bowel_sum~diabetes |
| Non-diabetic |  |  |  |  | bowel_sum~diabetes |
| Diabetic |  | -1.2 | -5.6, 3.2 | 0.6 | bowel_sum~diabetes |
| Drug allergy (any) | 1,783 |  |  |  | bowel_sum~allergy |
| FALSE |  |  |  |  | bowel_sum~allergy |
| TRUE |  | -1.1 | -3.2, 0.96 | 0.3 | bowel_sum~allergy |
| BMI | 1,765 | -0.06 | -0.22, 0.11 | 0.5 | bowel_sum~BMI |
| Radiotherapy dose (Gy) | 1,783 | 0.00 | -0.01, 0.01 | 0.7 | bowel_sum~totaldose |
| Treatment volume | 1,775 |  |  |  | bowel_sum~tx_vol |
| prostate bed |  |  |  |  | bowel_sum~tx_vol |
| pelvis |  | -0.85 | -2.8, 1.2 | 0.4 | bowel_sum~tx_vol |
| Race | 1,730 |  |  |  | bowel_sum~race |
| White |  |  |  |  | bowel_sum~race |
| Black |  | 0.61 | -1.4, 2.6 | 0.5 | bowel_sum~race |
| Other |  | 1.5 | -1.8, 4.8 | 0.4 | bowel_sum~race |

Supplemental table 3. Univariable general estimating equation analysis for urinary incontinence

| **Characteristic** | **N** | **Beta** | **95% CI** | **p-value** | test |
| --- | --- | --- | --- | --- | --- |
| Hemoglobin | 1,598 | -0.28 | -2.2, 1.6 | 0.8 | incont_sum~hg_count |
| White blood cell count | 1,596 | -0.86 | -2.0, 0.27 | 0.13 | incont_sum~wbc_count |
| Platelet count | 1,598 | 0.01 | -0.03, 0.05 | 0.8 | incont_sum~plt_count |
| Bladder V70Gy(%) | 1,755 | -0.27 | -0.46, -0.08 | 0.004 | incont_sum~bladder_v70_p |
| Bladder V65Gy(%) | 1,755 | -0.09 | -0.24, 0.07 | 0.3 | incont_sum~bladder_v65_p |
| Bladder V60Gy(%) | 1,441 | -0.10 | -0.27, 0.07 | 0.3 | incont_sum~bladder_V60_p |
| Bladder V50Gy(%) | 1,441 | -0.12 | -0.28, 0.03 | 0.11 | incont_sum~bladder_v50_p |
| Bladder V40Gy(%) | 1,755 | -0.10 | -0.22, 0.03 | 0.12 | incont_sum~bladder_v40_p |
| Age | 1,769 | -0.31 | -0.59, -0.03 | 0.030 | incont_sum~age |
| Time from RP to RT (mo) | 1,769 | -0.06 | -0.10, -0.01 | 0.021 | incont_sum~rp_rt_time |
| Anticoagulation use | 1,769 |  |  |  | incont_sum~anticoag |
| No |  |  |  |  | incont_sum~anticoag |
| Yes |  | -5.4 | -15, 4.1 | 0.3 | incont_sum~anticoag |
| Aspirin Use | 1,769 |  |  |  | incont_sum~aspirin |
| No |  |  |  |  | incont_sum~aspirin |
| Yes |  | -2.6 | -6.8, 1.5 | 0.2 | incont_sum~aspirin |
| Diabetes mellitus | 1,756 |  |  |  | incont_sum~diabetes |
| Non-diabetic |  |  |  |  | incont_sum~diabetes |
| Diabetic |  | -5.4 | -16, 4.8 | 0.3 | incont_sum~diabetes |
| Drug allergy (any) | 1,769 |  |  |  | incont_sum~allergy |
| FALSE |  |  |  |  | incont_sum~allergy |
| TRUE |  | 0.21 | -4.4, 4.8 | >0.9 | incont_sum~allergy |
| BMI | 1,751 | -0.04 | -0.46, 0.38 | 0.9 | incont_sum~BMI |
| Radiotherapy dose (Gy) | 1,769 | 0.01 | -0.01, 0.02 | 0.3 | incont_sum~totaldose |
| Treatment volume | 1,762 |  |  |  | incont_sum~tx_vol |
| prostate bed |  |  |  |  | incont_sum~tx_vol |
| pelvis |  | 0.69 | -3.2, 4.5 | 0.7 | incont_sum~tx_vol |
| Race | 1,716 |  |  |  | incont_sum~race |
| White |  |  |  |  | incont_sum~race |
| Black |  | -0.79 | -5.3, 3.7 | 0.7 | incont_sum~race |
| Other |  | 7.3 | -1.3, 16 | 0.10 | incont_sum~race |

Supplemental table 4. Univariable general estimating equation analysis for urinary irritation

| **Characteristic** | **N** | **Beta** | 95% CI | p-value | test |
| --- | --- | --- | --- | --- | --- |
| Hemoglobin | 1,523 | -0.14 | -1.1, 0.82 | 0.8 | irrit_sum~hg_count |
| White blood cell count | 1,520 | -0.53 | -1.2, 0.11 | 0.11 | irrit_sum~wbc_count |
| Platelet count | 1,523 | -0.02 | -0.04, 0.01 | 0.2 | irrit_sum~plt_count |
| Bladder V70Gy(%) | 1,670 | -0.09 | -0.19, 0.01 | 0.092 | irrit_sum~bladder_v70_p |
| Bladder V65Gy(%) | 1,670 | -0.03 | -0.10, 0.03 | 0.3 | irrit_sum~bladder_v65_p |
| Bladder V60Gy(%) | 1,370 | -0.04 | -0.10, 0.01 | 0.14 | irrit_sum~bladder_V60_p |
| Bladder V50Gy(%) | 1,370 | -0.05 | -0.10, 0.01 | 0.12 | irrit_sum~bladder_v50_p |
| Bladder V40Gy(%) | 1,670 | -0.01 | -0.08, 0.07 | 0.9 | irrit_sum~bladder_v40_p |
| Age | 1,685 | -0.09 | -0.23, 0.06 | 0.2 | irrit_sum~age |
| Time from RP to RT (mo) | 1,685 | -0.02 | -0.05, 0.00 | 0.069 | irrit_sum~rp_rt_time |
| Anticoagulation use | 1,685 |  |  |  | irrit_sum~anticoag |
| No |  |  |  |  | irrit_sum~anticoag |
| Yes |  | -2.8 | -6.2, 0.70 | 0.12 | irrit_sum~anticoag |
| Aspirin Use | 1,685 |  |  |  | irrit_sum~aspirin |
| No |  |  |  |  | irrit_sum~aspirin |
| Yes |  | 0.20 | -2.0, 2.4 | 0.9 | irrit_sum~aspirin |
| Diabetes mellitus | 1,670 |  |  |  | irrit_sum~diabetes |
| Non-diabetic |  |  |  |  | irrit_sum~diabetes |
| Diabetic |  | -0.40 | -4.6, 3.8 | 0.9 | irrit_sum~diabetes |
| Drug allergy (any) | 1,685 |  |  |  | irrit_sum~allergy |
| FALSE |  |  |  |  | irrit_sum~allergy |
| TRUE |  | -0.90 | -3.1, 1.3 | 0.4 | irrit_sum~allergy |
| BMI | 1,670 | -0.08 | -0.26, 0.09 | 0.4 | irrit_sum~BMI |
| Radiotherapy dose (Gy) | 1,685 | 0.00 | -0.01, 0.00 | 0.4 | irrit_sum~totaldose |
| Treatment volume | 1,677 |  |  |  | irrit_sum~tx_vol |
| prostate bed |  |  |  |  | irrit_sum~tx_vol |
| pelvis |  | 1.6 | -0.89, 4.0 | 0.2 | irrit_sum~tx_vol |
| Race | 1,633 |  |  |  | irrit_sum~race |
| White |  |  |  |  | irrit_sum~race |
| Black |  | -3.8 | -6.5, -1.1 | 0.006 | irrit_sum~race |
| Other |  | 1.1 | -2.3, 4.5 | 0.5 | irrit_sum~race |
| Tobacco Use | 1,684 |  |  |  | irrit_sum~tobhx |
| Never |  |  |  |  | irrit_sum~tobhx |
| Former |  | -0.58 | -2.7, 1.5 | 0.6 | irrit_sum~tobhx |
| Current |  | -5.1 | -9.4, -0.85 | 0.019 | irrit_sum~tobhx |

Supplemental table 5. Univariable general estimating equation analysis for sexual function

| **Characteristic** | **N** | **Beta** | | **95% CI** | **p-value** | t |
| --- | --- | --- | --- | --- | --- | --- |
| Hemoglobin | 1,461 | | -0.06 | -1.5, 1.4 | >0.9 | sexual_sum~hg_count |
| White blood cell count | 1,458 | | -0.37 | -1.3, 0.58 | 0.4 | sexual_sum~wbc_count |
| Platelet count | 1,461 | | 0.02 | -0.03, 0.06 | 0.5 | sexual_sum~plt_count |
| Age | 1,603 | | -0.25 | -0.50, 0.00 | 0.047 | sexual_sum~age |
| Time from RP to RT (mo) | 1,603 | | -0.02 | -0.07, 0.03 | 0.5 | sexual_sum~rp_rt_time |
| Aspirin Use | 1,603 | |  |  |  | sexual_sum~aspirin |
| No |  | |  |  |  | sexual_sum~aspirin |
| Yes |  | | 1.9 | -2.1, 5.9 | 0.4 | sexual_sum~aspirin |
| ADT delivered | 1,603 | |  |  |  | sexual_sum~htx_bin |
| No |  | |  |  |  | sexual_sum~htx_bin |
| Yes |  | | -4.0 | -8.5, 0.41 | 0.075 | sexual_sum~htx_bin |
| Diabetes mellitus | 1,589 | |  |  |  | sexual_sum~diabetes |
| Non-diabetic |  | |  |  |  | sexual_sum~diabetes |
| Diabetic |  | | -7.2 | -13, -1.6 | 0.012 | sexual_sum~diabetes |
| Drug allergy (any) | 1,603 | |  |  |  | sexual_sum~allergy |
| FALSE |  | |  |  |  | sexual_sum~allergy |
| TRUE |  | | 2.2 | -1.9, 6.2 | 0.3 | sexual_sum~allergy |
| BMI | 1,593 | | -0.10 | -0.44, 0.23 | 0.5 | sexual_sum~BMI |
| Radiotherapy dose (Gy) | 1,603 | | -0.01 | -0.02, 0.01 | 0.2 | sexual_sum~totaldose |
| Treatment volume | 1,595 | |  |  |  | sexual_sum~tx_vol |
| prostate bed |  | |  |  |  | sexual_sum~tx_vol |
| pelvis |  | | -3.9 | -8.3, 0.59 | 0.089 | sexual_sum~tx_vol |
| Race | 1,557 | |  |  |  | sexual_sum~race |
| White |  | |  |  |  | sexual_sum~race |
| Black |  | | 0.04 | -4.1, 4.2 | >0.9 | sexual_sum~race |
| Other |  | | 1.1 | -6.8, 9.0 | 0.8 | sexual_sum~race |

Supplemental table 6. Univariable analysis of predictors of grade 2+ GU toxicity

| **Characteristic**^1^ | **N** | **HR** | **95% CI** | **p-value** |
| --- | --- | --- | --- | --- |
| Age | 303 | 1.02 | 0.99, 1.05 | 0.2 |
| Race |  |  |  |  |
| White | 192 | — | — |  |
| Black | 84 | 1.49 | 0.98, 2.27 | 0.061 |
| Other | 19 | 0.91 | 0.36, 2.32 | 0.8 |
| Time from RP to RT (mo) | 303 | 1.00 | 1.00, 1.01 | 0.3 |
| Anticoagulation use |  |  |  |  |
| No | 283 | — | — |  |
| Yes | 20 | 2.37 | 1.22, 4.62 | 0.011 |
| Aspirin Use |  |  |  |  |
| No | 184 | — | — |  |
| Yes | 119 | 1.56 | 1.05, 2.31 | 0.027 |
| Tobacco use |  |  |  |  |
| Never | 140 | — | — |  |
| Previous | 123 | 1.15 | 0.74, 1.77 | 0.5 |
| Current | 39 | 1.44 | 0.82, 2.51 | 0.2 |
| Hormonal therapy |  |  |  |  |
| None | 81 | — | — |  |
| <1 year | 148 | 1.29 | 0.81, 2.06 | 0.3 |
| ≥1 year | 53 | 1.05 | 0.56, 1.97 | 0.9 |
| Diabetes mellitus |  |  |  |  |
| Non-diabetic | 279 | — | — |  |
| Diabetic | 20 | 1.28 | 0.65, 2.55 | 0.5 |
| Drug allergy (any) |  |  |  |  |
| None | 207 | — | — |  |
| Yes | 96 | 0.97 | 0.63, 1.48 | 0.9 |
| Body mass index (continuous) | 298 | 0.99 | 0.95, 1.04 | 0.7 |
| Treatment volume |  |  |  |  |
| Prostate bed | 83 | — | — |  |
| Pelvic Lymph nodes | 219 | 1.13 | 0.73, 1.75 | 0.6 |
| Hemoglobin | 267 | 0.90 | 0.76, 1.06 | 0.2 |
| White blood cell count | 266 | 1.01 | 0.90, 1.13 | 0.9 |
| Platelet count | 267 | 1.00 | 0.99, 1.00 | 0.4 |
| Bladder V70Gy (%) | 300 | 1.02 | 1.01, 1.04 | 0.008 |
| Bladder V40Gy (%) | 300 | 1.01 | 1.00, 1.03 | 0.081 |
| Bladder V30Gy (%) | 252 | 1.02 | 1.00, 1.03 | 0.033 |
| Bladder mean dose (Gy) | 252 | 1.00 | 1.00, 1.00 | 0.4 |
| Bladder max dose (Gy) | 147 | 0.97 | 0.82, 1.14 | 0.7 |
| ^1^All variables are evaluated continuously unless the levels are specified as above | | | | |
| Abbreviations: CI = Confidence Interval, HR = Hazard Ratio | | | | |

Supplemental Table 7. Univariable analysis of predictors of grade 2+ GI toxicity

| **Characteristic**^1^ | **N** | **HR** | **95% CI** | **p-value** |
| --- | --- | --- | --- | --- |
| Age | 303 | 1.00 | 0.94, 1.06 | 0.9 |
| Race |  |  |  |  |
| White | 192 | — | — |  |
| Black | 84 | 0.36 | 0.08, 1.64 | 0.2 |
| Other | 19 | 0.00 | 0.00, 0.00 | <0.001 |
| Time from RP to RT (mo) | 303 | 1.01 | 1.00, 1.02 | 0.017 |
| Anticoagulation use |  |  |  |  |
| No | 283 | — | — |  |
| Yes | 20 | 0.00 | 0.00, 0.00 | <0.001 |
| Aspirin Use |  |  |  |  |
| No | 184 | — | — |  |
| Yes | 119 | 1.57 | 0.55, 4.44 | 0.4 |
| Tobacco use |  |  |  |  |
| Never | 140 | — | — |  |
| Previous | 123 | 0.99 | 0.33, 2.95 | >0.9 |
| Current | 39 | 0.56 | 0.07, 4.56 | 0.6 |
| Hormonal therapy |  |  |  |  |
| None | 81 | — | — |  |
| <1 year | 148 | 0.78 | 0.25, 2.42 | 0.7 |
| ≥1 year | 53 | 0.30 | 0.04, 2.53 | 0.3 |
| Diabetes mellitus |  |  |  |  |
| Non-diabetic | 279 | — | — |  |
| Diabetic | 20 | 1.01 | 0.13, 7.73 | >0.9 |
| Drug allergy (any) |  |  |  |  |
| None | 207 | — | — |  |
| Yes | 96 | 0.92 | 0.29, 2.90 | 0.9 |
| Body mass index (continuous) | 298 | 0.97 | 0.86, 1.09 | 0.6 |
| Treatment volume |  |  |  |  |
| Prostate bed | 83 | — | — |  |
| Pelvic lymph nodes | 219 | 0.50 | 0.18, 1.43 | 0.2 |
| Hemoglobin | 267 | 0.84 | 0.58, 1.23 | 0.4 |
| White blood cell count | 266 | 0.92 | 0.70, 1.21 | 0.6 |
| Platelet count | 267 | 1.00 | 0.99, 1.01 | 0.6 |
| Rectum V70Gy (%) | 301 | 0.97 | 0.84, 1.12 | 0.7 |
| Rectum V65Gy (%) | 301 | 0.97 | 0.91, 1.03 | 0.3 |
| Rectum V40Gy (%) | 301 | 1.00 | 0.98, 1.02 | 0.8 |
| Rectum V30Gy (%) | 252 | 1.00 | 0.98, 1.03 | 0.8 |
| Rectum mean dose (Gy) | 252 | 1.00 | 1.00, 1.00 | 0.5 |
| ^1^All variables are evaluated continuously unless the levels are specified as above | | | | |
| Abbreviations: CI = Confidence Interval, HR = Hazard Ratio | | | | |
